# Jacks of metal/metalloid chelation trade in plants—an overview

**DOI:** 10.3389/fpls.2015.00192

**Published:** 2015-04-02

**Authors:** Naser A. Anjum, Mirza Hasanuzzaman, Mohammad A. Hossain, Palaniswamy Thangavel, Aryadeep Roychoudhury, Sarvajeet S. Gill, Miguel A. Merlos Rodrigo, Vojtěch Adam, Masayuki Fujita, Rene Kizek, Armando C. Duarte, Eduarda Pereira, Iqbal Ahmad

**Affiliations:** ^1^Centre for Environmental and Marine Studies and Department of Chemistry, University of AveiroAveiro, Portugal; ^2^Department of Agronomy, Faculty of Agriculture, Sher-e-Bangla Agricultural UniversityDhaka, Bangladesh; ^3^Department of Genetics and Plant Breeding, Bangladesh Agricultural UniversityMymensingh, Bangladesh; ^4^Department of Environmental Science, School of Life Sciences, Periyar UniversitySalem, India; ^5^Post Graduate Department of Biotechnology, St. Xavier's College (Autonomous)Kolkata, India; ^6^Stress Physiology and Molecular Biology Lab, Centre for Biotechnology, Maharshi Dayanand UniversityRohtak, India; ^7^Central European Institute of Technology, Brno University of TechnologyBrno, Czech Republic; ^8^Department of Chemistry and Biochemistry, Mendel University in BrnoBrno, Czech Republic; ^9^Laboratory of Plant Stress Responses, Faculty of Agriculture, Kagawa UniversityMiki-cho, Japan; ^10^Centre for Environmental and Marine Studies and Department of Biology, University of AveiroAveiro, Portugal

**Keywords:** metal/metalloids, plant tolerance, chelation, thiol compounds, glutathione, organic acid, metallothioneins, phytochelatins

## Abstract

Varied environmental compartments including soils are being contaminated by a myriad toxic metal(loid)s (hereafter termed as “metal/s”) mainly through anthropogenic activities. These metals may contaminate food chain and bring irreparable consequences in human. Plant-based approach (phytoremediation) stands second to none among bioremediation technologies meant for sustainable cleanup of soils/sites with metal-contamination. In turn, the capacity of plants to tolerate potential consequences caused by the extracted/accumulated metals decides the effectiveness and success of phytoremediation system. Chelation is among the potential mechanisms that largely govern metal-tolerance in plant cells by maintaining low concentrations of free metals in cytoplasm. Metal-chelation can be performed by compounds of both thiol origin (such as GSH, glutathione; PCs, phytochelatins; MTs, metallothioneins) and non-thiol origin (such as histidine, nicotianamine, organic acids). This paper presents an appraisal of recent reports on both thiol and non-thiol compounds in an effort to shed light on the significance of these compounds in plant-metal tolerance, as well as to provide scientific clues for the advancement of metal-phytoextraction strategies.

## Introduction

### Metal(loid)s and their chelation strategies in plants

The Earth's crust harbors varying levels of different metals/metalloids (hereafter termed as “metal/s”). Though at optimum level, many metals (such as Cu, Fe, Mn, Ni, Zn) are essential for plant cells; however, the supra-optimum concentrations of these metals and even low concentrations of other metals such as Ag, Al, As, Cd, Cr, Cs, Hg, Pb, Sr, and U exhibit phytotoxicity. Thus, higher concentrations of all metals that have potential to cause detrimental consequences in human or environments can be considered as “contaminant” (reviewed by Anjum et al., 2015). Nevertheless, the inception of industrialization, metalliferous mining and smelting, sewage sludge treatment, warfare, and military training, waste disposal sites and indiscriminate agricultural fertilizer use have caused significant addition of previous toxic metals to soils (Padmavathiamma and Li, 2007; Hassan and Aarts, 2011; Alloway, 2013). Though, it remained technically a challenge for the global scientific community, the cleanup of metal-contaminated soils has been widely advocated to minimize their impact on human and environmental health (reviewed by Ali et al., 2013). In this context, compared to different physical, chemical and biological approaches employed for this purpose, plant and associated microbes based approach (phytoremediation) stands outstanding in terms its novelty, cost-effective, efficiency, environment- and eco-friendly, *in situ* applicability, and natural (solar-driven) (Mench et al., 2009; Hassan and Aarts, 2011; Anjum et al., 2012a; Ali et al., 2013). Basically, the phytoremediation approach is based on a number of strategies including: (a) phytoextraction, (b) rhizofiltration (phytofiltration), and (c) phytostabilization. Notably, plant types growing on contaminated or metalliferous soils were evidenced to develop metal-hyperaccumulation potential (reviewed by Baker and Whiting, 2002). Metal-hyperaccumulation, a process technically termed as phytoextraction, is a striking phenomenon exhibited by <0.2% of angiosperms, where a direct accumulation of metals into above-ground organs with subsequent removal/ processing of these plant-organs is possible. Nevertheless, metal-hyperaccumulators can exhibit extraordinarily high amounts of metals in their above-ground tissues to levels far exceeding those present in the soil or in non-accumulating plant species growing nearby (reviewed by Hassan and Aarts, 2011; Rascio and Navari-Izzo, 2011; Gill et al., 2012).

**Figure 1:**
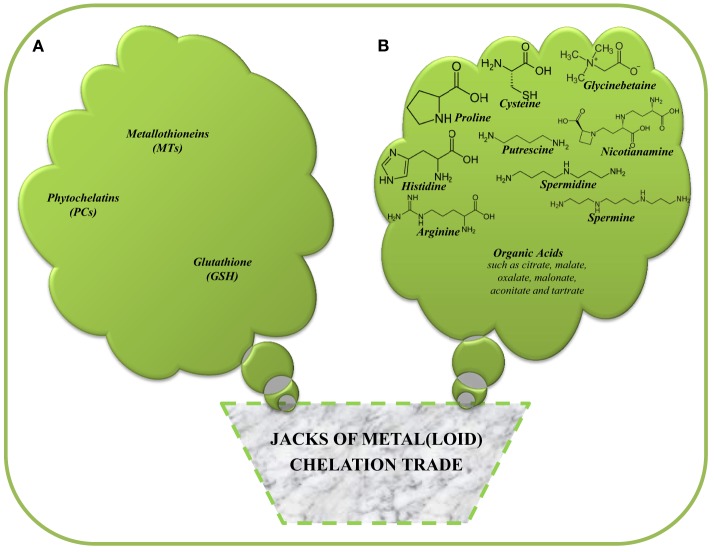
**Major thiol- (A) and non-thiol compounds (B) involved in the chelation/detoxification of metal(loid)s in plants**.

Understanding physiological and molecular defense strategies adopted by both hyperaccumulator and non-hyperaccumulator plants to cope with metal stress either during accumulation, degradation or elimination of metal pollutants in contaminated soils are of the utmost significance in phytoremediation studies. In particular, plant tolerance to potential toxicity caused by tissue-/organ-metal loads (during metal-hyperaccumulation/extraction) largely decides the efficiency and success of a metal remediation system (Vangronsveld et al., 2009; Maestri et al., 2010; Hassan and Aarts, 2011; Anjum et al., 2014a,b,c). Processes such as exclusion, compartmentalization, complexation, and the synthesis of metal-binding proteins and/or metal ion chelation are included in the list of defense strategies evidenced in plants under metal stress (Clemens, 2001; Mejáre and Bülow, 2001; reviewed by Hassan and Aarts, 2011). Plants have been credibly reported to avoid the damaging effects of metal toxicity, using strategies or mechanism involving: the binding of heavy metals to cell wall and immobilization (Mari and Lebrun, 2006; Kanneganti and Gupta, 2008; Bolan et al., 2014), exclusion of the plasma membrane (Lee et al., 2005; Arrivault et al., 2006), expression of more general stress response mechanisms such as stress proteins (heat shock proteins) (Song et al., 2012), and metal-chelation and -compartmentalization (Lal, 2010; Jozefczak et al., 2012; Seth et al., 2012; Lv et al., 2013). In particular, chelation is the most widespread intracellular mechanism for the maintenance of low concentrations and detoxification of free metals in plant cytoplasm that can be performed by thiol compounds (which contain sulfhydryl/thiol groups; such as a tripeptide glutathione, GSH, γ-Glu-Cys-Gly; phytochelatins, PCs; metallothioneins, MTs), and also by non-thiol compounds (such as organic acids, and amino acids) (Clemens, 2001; Mejáre and Bülow, 2001; Lal, 2010; Hassan and Aarts, 2011; Anjum et al., 2012b, 2014b,d; Jozefczak et al., 2012; Seth et al., 2012; Lv et al., 2013) (Figure 1).

Based on recent reports, this paper discusses the basic physiology/molecular biology of metal-chelation by GSH, PCs, MTs, organic acids, amino acids—considered herein as jacks of metal-chelation trade in plants.

## Thiol-compounds and metal(loid)-chelation

Thiol compounds such as GSH, PCs, and MTs contain sulfhydryl (–SH) groups for binding a variety of metals (reviewed by Seth et al., 2012). GSH, a tripeptide (γ-Glu-Cys-Gly) with a wide distribution (0.5–10 mM) in plant cell compartments (namely cytosol, endoplasmic reticulum, vacuole, and mitochondria) is a major –SH compound. Apart from performing key roles in cellular redox homeostasis and the antioxidant defense, GSH is involved in the chelation and detoxification of free metal (reviewed by Anjum et al., 2010, 2012a, 2014d; Seth et al., 2012). The role of GSH in metal-chelation lies behind its significance as a precursor for the synthesis of phytochelatins (PCs, family of peptides structurally related to GSH) in metal-exposed plants (Clemens, 2006; Srivalli and Khanna-Chopra, 2008). The other important –SH compound, MTs [sulfur(S)-containing, cysteine (Cys)-rich, short, low molecular weight (4–8 kDa) gene-encoded polypeptides] have been reported to bind varied metals through the –SH of their Cys-residues (Cobbett and Goldsbrough, 2002; Verbruggen et al., 2009). Therefore, GSH, PCs, and MTs stand second to none in terms of their role in metal-chelation in plants (Figure 1; Table 1). GSH is briefly overviewed hereunder before discussing other –SH compounds and S-donor ligands—PCs and MTs in detail.

**Table 1:** **Summary of representative studies on metal(loid)-tolerance in plants *via* expression of genes related with major thiol and non-thiol compounds**.

**Plant species**	**Gene inducing agent**	**Gene overexpressed**	**Defensive effects**	**Tolerance gained**	**References**
*Raphanus sativus L*.	Exogenous Spd (1 mM) under Cr stress	*RsADC* and *RsSPDS*	Increased biosynthesis of Put and Spd	Cr (VI) stress	Choudhary et al., 2012b
*Arabidopsis*	Induced by Pb stress	*At3g02470*	This gene encodes *S*-adenosylmethionine decarboxylase, an enzymes of PA biosynthesis pathway	Pb tolerance	Liu et al., 2009
*Raphanus sativus*	Exogenous Spd (1 mM) under Cu stress	*RsCOPT1* and *RsCOPT2*	Decrease of Cu uptake	Cu tolerance	Choudhary et al., 2012a
		*RsHMA5*	Decrease of Cu assimilation		
		*RsMT1C* and *RsCCH1*	Cu detoxification by regulating the levels of metallothionenins and Cu chaperones		
		*RsADC1*, *RsADC2*, and *RsSAMDC*	Regulated Put metabolism		
		*RsSPDS1* and *RsPAO2*	Regulated Spd metabolism		
		*RsSPDS3*, *RsPAO2*, and *RsPAO4*	Regulated Spm metabolism		
		*RsCYP79B3*, *RsYUC1*, and *RsYUC3*	Regulated IAA metabolism		
		*RsABA3*, *RsNCED*, *RsAAO3*, and *RsCYP707A3*	Regulated ABA metabolism		
*Arabidopsis halleri*	Zn (25, 50 μM)	*At5g19530*	Induction of Spm/Spd synthase family protein	Zn tolerance	Sharma and Dietz, 2006
		*At5g04610*	Induction of Spd synthase-related/Put aminopropyltransferase-related		
*Arabidopsis thaliana*	Cs (2 mM)	*At5g19530*	Induction of Spm/Spd synthase family protein	Cs tolerance	Sharma and Dietz, 2006
		*At1g80600*	Induction of acetylornithine aminotransferase, mitochondrial, putative/acetylornithine transaminase, putative/AOTA		
		*At5g46180*	Induction of Orn aminotransferase, putative/Orn-oxo-acid aminotransferase		
*Arabidopsis thaliana*	Pb(NO_3_)_2_ (25, 50 ppm)	*At5g19530*	Induced Spm/Spd synthase family protein	Pb tolerance	Sharma and Dietz, 2006
		*At1g23820*	Induced Spd synthase 1 (SPDSYN1)/Put aminopropyltransferase 1		
*Nicotiana tobacum*	150 μ M CdCl_2_	*TaMT3*	Increased the ability of ROS cleaning-up	Cd tolerance	Zhou et al., 2014
*Arabidopsis thaliana*	As exposure	*S1ptTECS*	Strong induction of GSH1 protein expression in the shoots	As tolerance	Li et al., 2006
*Arabidopsis thaliana*	Cd exposure	*S1ptTECS*	Three- to five-fold increase in γ-EC related peptides	Cd tolerance	Li et al., 2005
*Populus canescens*	0.01–0.1 mM ZnSO_4_	*GSH1*	Levels of GSH and PCs are maintained by GSH1 in these transgenics	Zn tolerance	Bittsánszkya et al., 2005
*Arabidopsis thaliana*	As exposure	*arsC*	Specific reduction of arsenate to arsenite in that easily trapped by thiols such as GSH and PCs	As tolerance	Dhankher et al., 2002
*Avicennia germinans*	Low concentration of Cd and Cu	*AvPCS*	Strong ROS scavenging activity in addition to high metal-binding capacity	Cd and Cu detoxification	Gonzalez-Mendoza et al., 2007
*Pyrus communis*	500 μM CuSO_4_	*MdSPDS1*	Alteration of polyamine titers in pear and reduction of Cu accumulation	Cu tolerance	Wen et al., 2008
*Arabidopsis thaliana*	Cu exposure	*MT1 and MT2*	Highest levels of non-protein thiols	Cu tolerance	Murphy and Taiz, 1995
*Saccharum* spp.	500μ M CdCl_2_ or 100μ M CuCl_2_	*ScMT2-1-3*	Enhanced Cu and Cd detoxification	Cu and Cd tolerance	Guo et al., 2013
*Alyssum lesbiacum*	30 μ M NiSO_4_	*ATP-PRT*	Many-fold increase in the pool of free His without affecting the concentration of any other amino acid	Ni tolerance	Ingle et al., 2005
*Arabidopsis halleri*	10 μ M ZnSO_4_	*NICOTIANAMINE SYNTHASE*s	Enhanced nicotianamine synthesis and subsequent binding of a variety of transition metals.	Zn tolerance	Haydon et al., 2012
*Arabidopsis halleri*	10 μ M ZnSO_4_	*NAS2*	Elevated nicotianamine levels and subcellular compartmentalization of a metal chelator in balancing the transport processes of Zn	Zn tolerance	Deinlein et al., 2012
*Nicotiana tabacum*	600 and 1000 mM of NiCl_2_	*AtNAS1*	Ten-fold elevated levels of NA in comparison with wild type which led to an enhanced tolerance against up to 1 mM Ni	Increased Ni tolerance	Douchkov et al., 2005

GSH is recognized as an antioxidant that plays a key role in the defense mechanism of plants (Anjum et al., 2010, 2012a, 2014d; Seth et al., 2012; Nahar et al., 2015). Notably, since GSH is also an essential component in the synthesis of metal-binding peptides such as PCs, GSH has been the major metabolic/biochemical modulator of of PCs (Hall, 2002; Guo et al., 2012). The ability to maintain a high GSH level is therefore considered as an essential intrinsic feature, enabling the reduction of oxidative damage caused by accumulated metals in these species. Some metal-tolerant plants, e.g., *Thlaspi goesingense*, *Thlaspi oxyceras*, *Thlaspi rosulare*, and *Holcus lanatus* have a constitutively high GSH content, unlike e.g., *Silene vulgaris* or *Pteris vittata* (Ernst et al., 2008). The higher levels of GSH and Cys were also found in Cd-resistant mutant of *Chlamydomonas reinhardtii* than the wild type (Hu et al., 2001), Cu-resistant alga *Stichococcus minor* (Kalinowska and Pawlik-Skowrónska, 2010) and 276-4d strains of *Desmodesmus armatus* (Pokora et al., 2014). Cys could act as a defense mechanism not only for participating in PC biosynthesis but also directly as metal-chelator. Cys content was higher in freshwater microalga *Chlamydomonas moewusii* than that of GSH when Cd was present in the medium. Further, the amount of GSH decreased only slightly with the increase in the PC levels. This would suggest that the intracellular GSH was consumed during the PC biosynthesis and replaced quickly by new synthesis from the S of the medium (Mera et al., 2014). Estrella-Gómez et al. (2012) showed that *Salvinia minima* plants responded to Pb exposure by increasing the concentration of GSH, the activity of glutathione synthase (GS) and the expression levels of the SmGS gene. Similarly, GSH and glutathione reductase (GR) levels were also increased when tomato *(Lycopersicon esculentum)* plants are exposed to arsenite in which the activity of GR is essential for recharging the cells of GSH and the time of increased synthesis of PCs (Marmiroli et al., 2014). On the other hand, since Glu and Cys are important precursors in GSH biosynthesis, it is somewhat surprising that proteins involved in Gln and Cys synthesis were less abundant upon long term Cd-exposure (Dupae et al., 2014). Cd-accrued PCs-induction can cause elevation in the consumption of GSH as a result of utilization of GSH [and also the γ-GluCys (γ-EC) moiety from GSH by transpeptidation] in the production of PCs (Mendoza-Cózatl et al., 2008). In contrast, elevation in GSH pool, reported in some metal-exposed plants has been considered as a strategy to detoxify/tolerate metals(Cd)-accrued consequences either by direct binding or by synthesis of PCs (Cánovas et al., 2004; Thangavel et al., 2007; reviewed by Anjum et al., 2012b). Although the concentrations of GSH in ectomycorrhizal fungus are even in millimolar range (Schützendübel and Polle, 2002; Courbot et al., 2004) and GSH readily binds both to Cd and Zn (Feretti et al., 2007), it may provide a fast protection against the metal ion transients entering the cytoplasm.

### Phytochelatins

#### Overview

Phytochelatins [PCs; γ-glutamyl (Glu)-cysteinyl (Cys)]*_n_*–X, where *n* = 2–11 and X is glycine (Gly), serine, β-alanine, glutamate or glutamine] are the principal non-protein, metal-binding (and metal-dotoxifying), S-rich, thiolate peptides (Cobbett and Goldsbrough, 2002). However, in iso-PCs (isoforms of PCs), the terminal amino acid consists of serine, glutamic acid, glutamine, or β-alanine (in the case of the homo-PCs, present in many legumes) (Oven et al., 2002). In fact, PCs are non-ribosomal peptides and are synthesized enzymatically (not by translation of mRNA owing to the presence of γ-carboxamide linkage between Glu and Cys) in response to varied metals from GSH by phytochelatin synthase (PCS), which is a γ-Glu-Cys dipeptidyl transpeptidase (E.C.2.3.2.15) (Vatamaniuk et al., 2004). Both GSH and hGSH can be the substrates for the synthesis of homo-PCs in Cd-exposed legume (Oven et al., 2002). Nevertheless, though, the amino acids Cys, Glu, and Gly constitute PCs (Zenk, 1996), PC variants without C-terminal Gly-residues can also be found (Oven et al., 2002). The occurrence of PC synthesis in metal-exposed plants was reported to follow different hierarchical levels from algae to higher plants including trees (Thangavel et al., 2007; Minocha et al., 2008). In earlier studies, PC production and the enzyme PCS were not found in land plants or bryophytes including liverworts, mosses, and hornworts (Bruns et al., 2001; Kopriva et al., 2007). *Micrasterias denticulata* is the only charophytic algae where the synthesis of PCs has until now been definitely detected (Volland et al., 2014). Recently, the PC-synthesis capability and the presence of constitutive and functional PCS were considered as ancestral (plesiomorphic) characters for basal land plants including bryophytes, charophytes, and lycophytes (Petraglia et al., 2014). A lower amount of PC produced under Cd stress (36 and 72 μ M of Cd for 72 h) in all the bryophyte lineages and in the lycophyte *Selaginella denticulataprevious* was suggested due to lower PCS activity as compared to angiosperms. Notably, potential toxicity of accumulated metals can be decreased as a result of the formation and subsequent sequestration of “metal-PC complexes” in vacuoles *via* transport across the tonoplast (Cobbett and Goldsbrough, 2002). Nevertheless, when expressed in an appropriate host the *Caenorhabditis elegans* PCS gene that control PCs production; however, knocking out the gene can increase the sensitivity of *C. elegans* to Cd (Vatamaniuk et al., 2001). Considering the previous roles of PCs as well as due to their significant role in the induction in plants under metal-exposure, the “status of PCs in plants” has been advocated as one of the major indicators of metal pollution (Dago et al., 2014).

#### Metal(loid)-specificity and -chelation mechanisms

Synthesis of PCs can be plant-specific and/or metal-specific. Depending on the metal type, Hg, Cd, As, Ag, and Fe have been reported as strong inducers of PCs while Pb and Zn are weak inducers and Cu and Ni are moderate inducers (Zenk, 1996). Among metals, Cd has been detected as a strong inducer of PCs in various plant species (Ortega-Villasante et al., 2005; Clemens, 2006; Rellán-Álvarez et al., 2006; Thangavel et al., 2007; Sobrino-Plata et al., 2009; Gill et al., 2012; Guo et al., 2012; Dago et al., 2014). Compared to Zn (Thangavel et al., 2007) and Hg (Ortega-Villasante et al., 2005; Rellán-Álvarez et al., 2006; Sobrino-Plata et al., 2009) Cd was observed as a major PC-inducer. Opposite to GSH, which was more concentrated in Hg-exposed *Hordeum vulgare* aerial parts than in *H. vulgare* roots, longer-chain PCs (such as PC3, PC4, and PC5) were more abundant in *H. vulgare* roots than in aerial parts of *H. vulgare* and were increased with increase in phytoavailable Hg in soils (Dago et al., 2014). Moreover, the decreased concentrations of smaller thiols such as GSH and PC2 with increasing phytoavailable Hg in soils can be due to use of both GSH and PC2 as substrates for the synthesis of the said longer-chain PCs. Pb and Cd exposure can cause the production of PC2 and PC4, respectively, in *Phaeodactylum tricornutum* cells (Morelli and Scarano, 2001). Among different chains of PC synthesis (PC2–PC5), PC3 and PC4 were the major PCs exhibited in Cd-exposed microalga *C. moewusii* (Mera et al., 2014). Similarly, PC3 was the major peptide in both the 276-4d and B1-76 strains of green alga *Desmodesmus armatus* under 93 μ M Cd (Pokora et al., 2014). However, the other PC-oligomers (PC2, PC4 and unidentified P1, P2, and P3) were also higher in B1-76 strain in the first-phase of cell cycle. The unidentified thiol P1, found in green alga *Stichococcus bacillaris* may represent γ-Glu-Cys as the precursor of GSH and PC synthesis (Pawlik-Skowrónska, 2002). The remaining two unidentified non-protein thiols P2 and P3 were also found in the freshwater green alga *Stigeoclonium tenue* that differed from each other in one γ-Glu-Cys unit and contained an additional Cys-residue which was resistant to a mixture of heavy metals (Pawlik-Skowrónska, 2003). Although a linear relationship between Cd and PC production was previously observed in numerous algal species (Gekeler et al., 1988), the amount of synthesized PC after Cd-exposure may not reflect exactly the level of Cd-accumulation (Ahner et al., 1995; Nishikawa et al., 2006). Further, the mechanism underlying the Cd-efficiency for PC synthesis-activation remained unproven. PCs can also function as important chelators of Zn ions (Tennstedt et al., 2009). Song et al. (2014) suggested that essential metal ions, such as Zn(II), Cu(II), and Mn(II), can be transported into vacuoles as forms of “PC2-metal complexes” through the putative ABC transporter(s). However, the efficiency and speed of free Zn^2+^ chelation in the cytoplasm was higher in Cd/Zn hyperaccumulator *Arabidopsis halleri* than in *Arabidopsis thaliana* and these results are helpful to identify the metal sensitivity of the plants in terms of changes in the plasma membrane potential of root cortical cells (Ovečka and Takáč, 2014).

As highlighted also above that the –SH group of the Cys-residues help PCs to bind and generate strong “PC-metal complexes” in high metal-exposed plants. Subsequently, the “PC-metal complexes” are sequestered into vacuoles (via ABC type transporters, Verbruggen et al., 2009 or a group of organic solute transporters, Solanki and Dhankhar, 2011) for detoxification. Several studies have provided a strong evidence for the formation of Pb-PC complex and its role in Pb tolerance in plants (Zhang et al., 2008; Andra et al., 2010; Fernández et al., 2012). Spisso et al. (2014) also found Hg-PC complexes especially Hg-PC2, Hg-PC3, and Hg-PC4 in *Vitis vinifera* under 100 mg L^−1^ of Hg-exposure. The PC-As (III) complexation in rice leaves was reported to reduce translocation of As from leaves to grains (Duan et al., 2011). The chelation of Cd with PCs in the cytoplasm and compartmentalization of the PC-Cd complexes in the vacuole are generally considered as a “first line” of defense mechanism against Cd phytotoxicity (Inouhe, 2005). The PC-Cd complexes are up to 1000 times less toxic to many enzymatic proteins than the free Cd ions (Solt et al., 2003). The increase in the cytosolic pH of aquatic macrophyte *Elodea canadensis* after Cd addition could contribute to symplasmic Cd detoxification through PC-Cd complex formation (Tariq Javed et al., 2014), because PC-Cd complex stability increases with a rise in pH (Dorcak and Krezel, 2003). Further, such increase in cytosolic pH also activates vacuolar transporters as reported for *Saccharomyces cerevisiae* (Park et al., 2012). Mendoza-Cózatl et al. (2008) identified high concentrations of PCs, GSH, and Cd in the phloem sap of *Brassica napus* and suggested that, along with the xylem, the phloem is a channel for long-distance source-to-sink transport of Cd-PC and Cd-GSH complexes.

In contrast to the facts discussed above, overexpression of PCS gene does not always have beneficial effects on heavy metal tolerance. The heterologous overexpression of *Triticum aestivum TaPCS1* in rice increased Cd sensitivity and significantly increased Cd accumulation in shoots but not in roots (Wang et al., 2012). On the contrary, expression of *CdPCS1* from aquatic macrophyte *Ceratophyllum demersum* in tobacco (*Nicotiana tabacum), Escherichia coli* (Shukla et al., 2012) or *Arabidopsis* (Shukla et al., 2013) enhanced PC synthesis as well as Cd and As accumulation. As observed in the previous studies mentioned earlier in this section, higher level of PC accumulation was also detected in roots of cucumber (*Cucumis sativus*) plants treated with 25 or 50 μ M Cd, individually or simultaneously with selenium (Se) (Hawrylak-Nowak et al., 2014). PC4 and PC2 were predominant in individual Cd-exposed cucumber roots and leaves, respectively. However, a reduction of PCs accumulation was observed only in roots of cucumber when Cd-exposed plants with Se addition; whereas no change in GSH and PC contents in leaves (Hawrylak-Nowak et al., 2014). Because Se interferes with S metabolism and can replace S in the S-amino acids which results into the production of the corresponding Se-amino acids (seleno-Cys and selenomethionine), their subsequent incorporation into enzymatic proteins may affect catalytic activity (Ellis and Salt, 2003). The catalytic moiety of PCS enzyme contains the active Cys. Moreover, the C-terminal domain of PCs from different species shows low sequence conservation, but shares a common feature in that they all contain multiple Cys residues that bind Cd ions with high affinity and high capacity (Wang et al., 2009). Thus, the replacement of Cys by seleno-Cys in the PCs probably may affect the biosynthesis and accumulation of PCs in the plant tissues (Hawrylak-Nowak et al., 2014).

### Metallothioneins

#### Overview

Another important metal chelator –SH compound and S-donor ligand, metallothioneins (MTs) are products of mRNA translation, characterized as low mass weight (4–14 kDa) Cys-rich metal-binding proteins, and are widely distributed in both prokaryotic and eukaryotic organisms (Cobbett and Goldsbrough, 2002). Margoshes and Vallee (1957) for the first time characterized MTs from horse kidneys as Cd-binding proteins. Based on sequence similarities and phylogenetic relationships plant MTs are divided into four subfamilies, type 1–4 (Freisinger, 2008; Hassinen et al., 2011). Type 1 and 2 sequences contain two Cys-rich domains separated by a central Cys-free spacer. In type 1 sequences the Cys residues are exclusively arranged in Cys-Xaa-Cys motifs (Xaa represents another amino acid) in both the N- and C-terminus, while type 2 have Cys-Cys, Cys-Xaa-Xaa-Cys, and Cys-Xaa-Cys sequences in the N-terminal domain and Cys-Xaa-Cys in the C-terminal domain. Each type of MT genes displays a distinct spatial and temporal expression pattern (Cobbett and Goldsbrough, 2002; Hassinen et al., 2011). Based on structural models, it can be assumed that the MT molecule is composed of two binding domains, α and β, which are composed of Cys-clusters. Covalent binding of metal atoms involves sulfhydryl cysteine residues. The N-terminal part of the peptide is designated as β-domain and has three binding sites for divalent ions, and the C-terminal part (the α-domain) has the ability to bind four divalent metal ions (Ruttkay-Nedecky et al., 2013).

#### Metal(loid)-specificity and -chelation mechanisms

MTs exhibit their high affinity for both essential and non-essential metals, where MTs can provide thiols for metal chelation in their reduced state. High affinity of MTs for metals provides not only a mechanism for protection against the toxicity of different metals (such as Cd) but it is also significant for the maintenance of homeostasis of some essential metals such as Zn and Cu ions. A general structure for plant MT has been proposed for *Triticum durum* MT type I. This protein forms dimers or higher oligomers and adopts an extended conformation containing both α-helix and β-sheet structures. This model proposes an overall dumbbell shape similar to that reported for mammalian MTs (Bilecen et al., 2005), in agreement with dynamic data obtained on *Fucus vesiculosus* MTs (Merrifield et al., 2006). The involvement of peptide donor groups (S-thiol and N-imidazole) and non-protein ligands (as sulfide anions) in metal chelation, as well as secondary structure elements, was demonstrated. The protein accommodates up to six Cd^2+^ together with four S^2−^; while, less than four Zn^2+^ could bind the protein (Zimeri et al., 2005). The combination of high thermodynamic but low kinetic stability is one of the main features of the metal-MT complexes, which bind the metals very tightly but a part of the metal ions is easily exchanged for other proteins (Hassinen et al., 2011). The MT superfamily combines a large variety of small Cys-rich proteins that have the ability to coordinate various transition metal ions, including Zn^2+^, Cd^2+^, and Cu^+^ (Freisinger, 2011). Metal ions can also be coordinated through His residues, but the impact of this ligand on the metal binding properties and function of MTs is not clear(Blindauer, 2008).

There exists inconsistency in the literature available on major stimuli capable of MTs induction/expression in plants. Though abiotic stresses such as drought, salinity, heat, cold light, wounding and senescence can modulate MT gene expression in plants (references cited in Sekhar et al., 2011), among metals, Cu, Cd, Pb, and Zn can strongly induce the plant MT gene expression (reviewed by Mehes-Smith et al., 2013). Cu-induced expression of a Type 1 MT gene in *Arabidopsis*. Guo et al. (2003) were the first to describe the expression of the complete MT gene family in *Arabidopsis* and their responses to Cu treatment. In non-accumulator plants, like *A. thaliana*, *MT1a* and *MT1b* are expressed at high levels in roots during exposure to Cd, Cu, and Zn (Maestri et al., 2010). In *Thlaspi caerulescens*, the levels of MT1mRNA were found in leaves constitutively higher than in roots; the levels increased with exposure to Cu. The primary sequence of the type 3 MT of *T. caerulescens* displays modifications that are proposed to increase its Cu-binding properties when compared to the non-hyperaccumulator, *A. thaliana* ortholog. *A. halleri* and *T. caerulescens* have a constitutively high expression of *MT2*. The expression of *MT3* genes increases during leaf aging and upon exposure to Cu in non-accumulator plants (Roosens et al., 2004). *MT4* is highly expressed in seeds of *A. thaliana*. It plays a role in metal homeostasis during seed development and seed germination rather than in metal decontamination (Roosens et al., 2004; Maestri et al., 2010). The analysis of the expression of *MT2a* in *A. thaliana*, a non-Pb tolerant species, showed that were specifically over-expressed in roots by Pb-treatment (Auguy et al., 2013). MTs are extremely diverse in plants and *T. caerulescens* seems to be an excellent model to understand the adaptive significance of this phenomenon. MT1, MT2, and MT3-related cDNAs were isolated in *T. caerulescens* and both *TcMT1*- and *TcMT3*-deduced protein sequences display modifications in their Cys domains when compared to their homologs in *A. thaliana*. Functional tests in yeast indicated that such modifications may alter the metal chelation of the plant MT proteins. Roosens et al. (2005) showed that the drastic decrease in the Cys number of domain 1 of *TcMT1* is associated with a lower tolerance to Cd and Zn of the yeast expressing *TcMT1* when compared to *AtMT1*. Ectopic expression of MT1 and MT2 (from *Brassica campestris*) in *A. thaliana* enhanced the tolerance to Cd and Cu and increased the Cu concentration in the shoots of the transgenic plants. Transgenic *Arabidopsis* accumulated less reactive oxygen species (ROS) than wild-type plants. *BcMT1* and *BcMT2* increased Cd and Cu tolerance in transgenic *Arabidopsis*, and decreased production of Cd- and Cu-induced ROS, thereby protecting plants from oxidative damage (Lv et al., 2013). The expression levels of the genes *MT2a*, *MT2b*, and *MT3* showed to be much higher in *T. caerulescens* than in non-metallophyte, non-hyperaccumulating reference species, as shown by microarray analyses. The expression of *MT2a* and *MT2b* in the roots is much higher in *T. caerulescens* than in *A. thaliana* (van de Mortel et al., 2006). Various MTs involved for As detoxification in *Oryza sativa* was also reported (Gautam et al., 2012). The authors have also shown that 11 class I MT genes in rice genome that are expressed differently during the growth and development (Gautam et al., 2012) which is influenced on Cu and Cd tolerance in plants (Zhou and Goldsbrough, 1994). *Arabidopsis* plants overexpressing pigeon pea *CcMT1* were more tolerant to Cu and Cd (Sekhar et al., 2011), while the garlic MTs *AsMT2b* and the *Colocasia esculenta* protein CeMT2b simultaneously confer Cd tolerance and promote Cd accumulation (Zhang et al., 2006). MT-like protein has also been found in *Chlorella vulgaris* capable of detoxifying Cd and Zn toxicities (Huang et al., 2009a). Recently, Zn (100 μ M L^−1^) was reported cause a higher induction of Zn-MT-like proteins (1.65-fold than control) in *C. vulgaris* (Yang et al., 2014). In a recent study by Nath et al. (2014), *MT1* and *MT2* has strongly expressed in *O. sativa* during 5 days of As (V) exposure. The ability of MTs to bind and sequester with metals/metalloids depends upon the distribution and organization of Cys residues and their regulated expression during stress is a major option for metal detoxification and homeostasis (Usha et al., 2009; Singh et al., 2011; Gautam et al., 2012).

## Non-thiol compounds and metal(loid)-chelation

**Figure 2:**
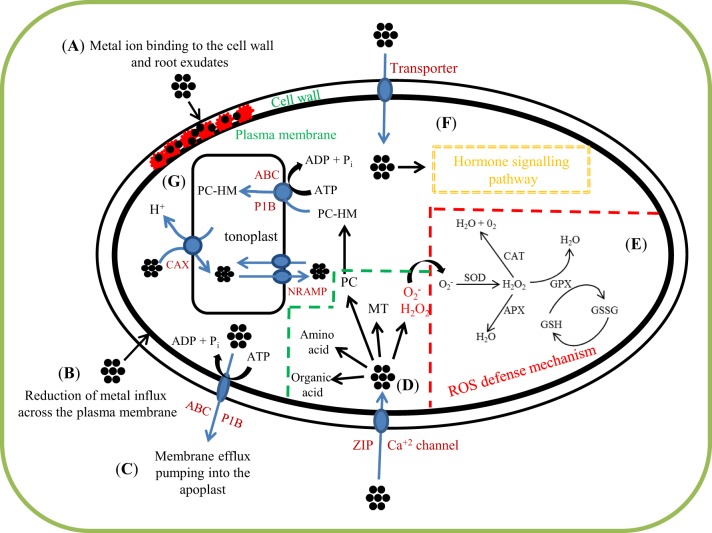
**Schematic representation of major functions, interrelationships among thiol and non-thiol compounds, and their coordination with other defense system components in metal(loid)-exposed plants**. As discussed in the text, plant responses to heavy metals include: **(A)** metal ion binding to the cell wall and root exudates; **(B)** reduction of metal influx across the plasma membrane; **(C)** membrane efflux pumping into the apoplast (ATP-binding-cassette (ABC) and P_1B_-ATPase transporter); **(D)** heavy metal (HM) chelation in the cytosol by ligands such as phytochelatins (PC), metallothioneins (MT), organic acids, and amino acids; **(E)** ROS defense mechanism [Antioxidant enzymes (SOD: superoxide dismutase, CAT: catalase, APX: ascorbate peroxidase, GPX: glutathione peroxidase, GSH: glutathione reduce and GSSG: glutathione oxidase)]; **(F)** hormone signaling pathway. **(G)** Transport and compartmentalization in the vacuole (ABC and P1B-ATPase transporter, NRAMP: natural resistance associated macrophage protein, CAX: cation/proton exchanger). Metal ions are shown as black dots.

A number of non-thiol compounds such as organic acid (OAs, including citrate, malate, oxalate, malonate, aconitate, and tartrate) and amino acids and their derivatives (including glycinebetaine/betaine; proline, Pro; histidine, His; cysteine, Cys; arginine, Arg; glutamate, Glu; nicotianamine, NA) in isolation and/or in coordination with thiol compounds have been credibly evidenced to contribute to metal-chelation in plants (Hall, 2002; Sharma and Dietz, 2006; Jakkeral and Kajjidoni, 2011) (Figure 2). Hereunder follows an appraisal of recent studies on chelation of metals in plants considering the mentioned above major non-thiol compounds.

### Organic acids

#### Overview

Organic acid (OAs) such as citrate, malate, oxalate, malonate, aconitate, and tartrate are low molecular weight weak-acidic compounds, possess at least one carboxyl group, and are termed as oxygen-donor metal ligands. Some of these compounds are present in all plant cells as intermediates of the tricarboxylic acid cycle (TCA), the main respiratory pathway involved in the oxidation of pyruvate (Trejo-Tellez et al., 2012). OAs potentially perform multiple functions in the rhizosphere. Contingent to the number of carboxylic groups and dissociation properties, OAs can carry varying negative charge, thereby allowing the complexation of metal cation in solution and displacement of anions from the soil-matrix (Jakkeral and Kajjidoni, 2011). Present in considerable amounts, citrate belongs to the key metabolites in plant cells. Lemons, wild strawberries and spinach leaves contain from 8 to 15% citrate, based on dry weight (Popova and Pinheiro de Carvalho, 1998). Metabolism of citrate in plants is carried out by several metabolic pathways located in different cellular compartments. Citrate has been extensively reported to be involved in carbon metabolism (as an intermediate) and plant-tolerance to varied stresses of abiotic (metals, nutrient deficiencies) and biotic (plant-microbe interactions operating at the rhizosphere) types (reviewed by Trejo-Tellez et al., 2012). Oxalate, C2 dicarboxilic acid anion is one of the strong OAs and a common constituents of plants. Though oxalate can be found in relatively small amounts in plants, it can be accumulated at high levels in several plants species (called extreme oxalate accumulators; can exhibit >3–18% oxalate based on their dry weight) from the families Caryophyllaceae, Chenopodiaceae, and Polygonaceae (Massey, 2003). Since oxalate can combine with various plant ions to from soluble or insoluble compounds, oxalate has been suggested to balance the excess of various inorganic cations (such as K^+^, Na^+^, NH^+^_4_, Ca^++^, and Mg^++^) over anions (such as NO^−^_3_, Cl^−^, H_2_PO^−^_4_, and SO^2−^_4_) (reviewed by Çalişkan, 2000). The role of oxalic acid in Al-tolerance has been well-documented (Furukawa et al., 2007; Liu et al., 2009; Maron et al., 2010; Yokosho et al., 2010). The dicarboxylic acid malate (an intermediate of the TCA cycle), an important plant metabolite, can be present in all cell types, and accumulated in plants to the levels up to 350 mM concentration. Malate in plants is involved in photosynthesis (C3, C4, and CAM plants), respiration and energy metabolism, fatty acid oxidation, stomatal, and pulvinual movement, lignin biosynthesis, nitrogen fixation, amino acid biosynthesis, ion balance, P- and Fe-uptake, and Al-tolerance (reviewed by Finkemeier and Sweetlove, 2009).

#### Metal(loid)-specificity and -chelation mechanisms

Metal-complexation with OAs has been argued to have potential role in the long distance xylem transport of heavy metals (Rascio and Navari-Izzo, 2011). Among the various OAs studied in plants, citrate has a high capacity to chelate metal ions and has been well-documented in the case of Fe and Al (Clemens, 2001; Singh and Chauhan, 2011). However, other metals such as Zn, Co, Ni, and Cd also exhibit their strong affinity for citrate. Cd-citrate complexes were evidenced in the xylem sap of *Arabidopsis hallerii* (Ueno et al., 2008). Involvement of citrate was evindenced in Cu-exclusion mechanisms in non-accumulators (reviewed by Mehes-Smith et al., 2013) and Ni-exclusion (Hall, 2002). The role of Ni-complexation with citric acid in Ni-uptake and hyperaccumulation has been reported (Boominathan and Doran, 2003). Although exudation of OAs is common Al-tolerance mechanism in different plant species, there are species-specific peculiarities worth noting (Simões et al., 2012). In general, citrate has the maximum ability followed by malate and oxalate to alleviate Al-toxicity (Singh and Chauhan, 2011). The mechanism of Al-tolerance in *Sorghum bicolor*, *Glycine max*, *Zea mays*, and *Hordeum vulgare* involves mainly citrate exudation/release (Furukawa et al., 2007; Maron et al., 2010). Similarly, citrate exudation has also been found to contribute to Al-tolerance in *T. aestivum*, *Arabidopsis*, and rye (Liu et al., 2009; Yokosho et al., 2010). In *O. sativa*, citrate exudation (Yokosho et al., 2011) as well as symplastic mechanisms are likely to contribute to the extreme Al-tolerance in this species (Huang et al., 2009b). A correlation between citric acid exudation and Al-tolerance was detected by Miyasaka et al. (1991) in *Phaseolus vulgaris*. Co-occurrence of different Al-tolerance mechanisms has also been reported in some species. In *Z. mays*, root oxalate (Kidd et al., 2001) and citrate (Piñeros et al., 2002) exudation are likely involved in Al-tolerance. However, Piñeros et al. (2005) observed a low correlation between citrate exudation and Al tolerance in *Z. mays*, suggesting that this species has other complementary mechanisms enabling them to tolerate Al stress. Exposure of cells of the Co-hyperaccumulator *Crotalaria cobalticola* and non-accumulators *Raufolia serpentina*, and *Silene cucubalus* to Co-ions resulted in an increase of citrate, indicating the involvement OA in the complexation of metal ions (Oven et al., 2002). The Al-activated mechanism of malate exudation is well-described in a number of plants including *B. napus* (Ligaba et al., 2006), *A. thaliana* (Hoekenga et al., 2006), *T. aestivum* (Sasaki et al., 2004), and rye (*Secale cereale*) (Collins et al., 2008). Recently, Zhu et al. (2011) showed that Cd-induced oxalate secretion from root apex is associated with Cd exclusion and resistance in *L. esculentum*.

Considering mechanisms underlying OAs-assisted metal-chelation, OAs confer metal-tolerance by transporting metals through the xylem and sequestrating ions in the vacuole, but they have multiple additional roles in the cell (reviewed by Fernie and Martinoia, 2009; Finkemeier and Sweetlove, 2009; Yang et al., 2013). Mechanism of metal-tolerance and detoxification in plants can be divided into two categories: external exclusion and internal tolerance. In the external detoxification process, organic acids excreted from plant roots may form stable metal–ligand complexes with metal ion and change their mobility and bioavailability, thus preventing the metal ions from entering plants or avoiding their accumulation in the sensitive sites of roots. In internal metal-detoxification, OAs may chelate with metal in the cytosol, where the ions can be transformed into a non-toxic or less toxic form (Clemens, 2001; Hall, 2002). The chelation of metals with ligands, such as OAs, amino acids and thiols facilitates the movements of heavy metals from roots to shoots (Zacchini et al., 2009). The xylem cell wall has a high cation exchange capability, thus the movement of metal cations is severely retarded when the metals are not chelated by ligands. OAs are involved in the translocation of Cd in the species *Brassica juncea* (Salt et al., 1999). OA-mediated Al stress tolerance has been well-studied in plants (Ma et al., 2001; Singh and Chauhan, 2011; Delhaize et al., 2012).

### Amino acids and their derivatives

#### Overview

Due to metal-binding capacity, amino acids and their derivatives may be deployed in response to metal-toxicity and in conferring to plants resistance to toxic levels of metal ions (Manara, 2012). However, a clear correlation between metal accumulation and the production of these compounds has not been established yet. Some of the amino acids, e.g., glycinebetaine (betaine), proline (Pro), histidine (His), cysteine (Cys), arginine (Arg), glutamate (Glu), nicotianamine (NA), and the polyamines (spermidine, Spd; spermine, Spm; putrescine, Put), are synthesized in the small (milimollar range) amount in response to metal stress (Figure 1). Thus, in many cases, nitrogen (N) metabolism is vital to the response of plants to metals (Sharma and Dietz, 2006). Based on several studies on different plants, chelation of metals by previous compounds and subsequent compartmentalization of the complexes formed with varied metals are well-established mechanisms for the detoxification of and tolerance to excess metals in plants (Hall, 2002; Sharma and Dietz, 2006).

Betaines are quaternary ammonium compounds (QACs) which contain a carboxylic acid group. They may be generally regarded as fully N-methylated amino or imino acids. There are several kinds of betaine among which glycine betaine (GB) is the most common. GB, also called as original betaine (N,N,N-trimethylglycine) was first discovered from *Beta vulgaris* which is later found to be distributed in microorganisms, plants and animals. It is one of the most abundant quaternary ammonium compounds those are accumulated in plants during dehydration-promoting conditions (Ashraf and Foolad, 2007). The role of GB as a significant osmoprotectant, ROS-scavenger, and metal-chelator has been reported in metal-exposed plants (Sharma and Dietz, 2006; Theriappan et al., 2011; Asgher et al., 2013; Kumchai et al., 2013; Gill et al., 2014). The other non-proteinogenic amino acid nicotianamine (NA) is ubiquitous in higher plants, and is considered as a key element in plant metal chelation and homeostasis (Takahashi et al., 2003; Rellán-Álvarez et al., 2008). The first step of NA biosynthesis is the formation of *S*-adenosylmethionine (SAM) from methionine by the action of *S*-adenosylmethionine synthetase (SAMS) (Mori et al., 2007). Arginine (Arg) is the most functionally diverse amino acid in living cells. Thus, role of Arg has come into light due to its multiple metabolic fates. Apart from serving as a constituent of proteins, Arg is a precursor for biosynthesis of other substances like polyamines (PAs), agmatine and Pro, Glu and nitric oxide (NO) those play vital role in metal stress tolerance (Liu et al., 2006; Nasibi et al., 2013). Arg biosynthesis in plant occurs through ornithine (Orn) which comes from few steps conversion of Glu involving five enzymes (Verma and Zhang, 1999). These compounds including NA, PAs, Pro, Glu, and NO play vital role in metal chelation and detoxification in plants (Hasanuzzaman and Fujita, 2013; Hasanuzzaman et al., 2013, 2014).

PAs are ubiquitous low molecular weight polycationic aliphatic amines have roles in plant growth, development and senescence. Diamine Put [NH_2_(CH_2_)_4_NH_2_], triamine Spd [NH_2_(CH_2_)_3_NH(CH_2_)_4_NH_2_], tetramine Spm [NH_2_(CH_2_)_3_NH(CH_2_)_4_NH(CH_2_)_3_NH_2_] are most common PAs in higher plants which exist as soluble conjugated, and insoluble bound forms and differential forms of PAs function differently (Lefevre et al., 2001). Some other PAs homospermidine, 1,3-diaminopropane, cadaverine, and canavalmine also common in some plants, animals, algae, and bacteria (Valero et al., 2002). Diversified properties of PAs including acid neutralizing, antioxidant properties, membrane and cell wall stabilizing abilities make those potent protectants against environmental stresses (Zhao and Yang, 2008). Both endogenous PAs and exogenous application of PAs confer tolerance against different stresses including metals (Groppa et al., 2003; Wang et al., 2007; Hasanuzzaman et al., 2014).

#### Metal(loid)-specificity and -chelation mechanisms

Literature is scarce on the specificity of amino acids and their derivatives to varied metals. However, plants exposed to varied metals (such as Cd, Cu, Ni, and Zn) can accumulate and synthesize different N-containing metabolites including Pro, amino acids, and oligopeptides, betaine, PAs, and NA. Pro and His have metal-binding, antioxidant, and signaling functions and can be accumulated in plants in response to different metals including As, Cd, Cu, Hg, and Ni (Kerkeb and Krämer, 2003; Sharma and Dietz, 2006; Irtelli et al., 2009; Richau and Schat, 2009; Richau et al., 2009; Theriappan et al., 2011; Ahmad and Gupta, 2013; Anjum et al., 2014b; Gill et al., 2014). NA and His/Pro were among the most important Cu-chelators in xylem sap of *Brassica carinata* under conditions of Cu deficiency and excess, respectively (Irtelli et al., 2009). Elevated accumulation of NA was evident different plants exposed to a number of metals such as Cd, Cu, Fe, Ni, and Zn (Takahashi et al., 2003; Vacchina et al., 2003; Kim et al., 2005; Irtelli et al., 2009; Kawachi et al., 2009; reviewed by Hassan and Aarts, 2011 and Mehes-Smith et al., 2013). In fact, the presence of six functional groups in NA allows its octahedral coordination and an optimal structure ideal for chelation of metal ions (Rellán-Álvarez et al., 2008). Notably, the pK of the resulting “metal-NA complexes” as well as the pH of the solution (with most metals being chelated at neutral or basic pH values) were considered to significantly control the ability of NA to chelate metals (Rellán-Álvarez et al., 2008). Apart from acting as an antioxidant, PAs function mainly as signaling molecule and can activate metal detoxification mechanisms (Sharma and Dietz, 2006). PAs are strongly supported to stabilize and protect membrane from toxic effects of metal ions specially the redox active metals (Sharma and Dietz, 2006). As a cation PAs can mimic and compete for the binding with Mg^2+^ and Ca^2+^ on receptors, membranes and enzymes. Polyamines bind cations including Cu, Fe that protects cell. The roles of PAs as metal chelators are contradictory. But some other N-containing compounds like porphyrins and chlorophylls strongly bound Cu, Fe, Co, and Ni by forming complexes. Presences of complex groups in same molecule regulate chelation effects. That is why complexes of PAs with high number of N-groups supposed to enhance chelation mechanism (Løvaas, 1997).

Amino acids and their derivatives exhibit their affinity to different metals, play vital role in their chelation and subsequently confer metal stress tolerance in plants. However, potential mechanisms underlying amino acids and their derivatives-assisted metal-chelation are not so conclusive in the literature. Both Pro and GB are potential osmoprotectants and they are mostly studied in plants grown under salinity and drought stress. However, role of Pro and GB in osmoprotection, ROS-scavenging, and metal-chelation has also been studied in many metal-exposed plants (Sharma and Dietz, 2006; Theriappan et al., 2011; Asgher et al., 2013; Kumchai et al., 2013; Gill et al., 2014). In addition, involvement of the elevated level of Pro in regulation of expression of genes of PCS, metallothionine-2 (MT-2), glutathione reductase (GR), and glutathione synthetase (GS) was recently reported in As-exposed plants (Ahmad and Gupta, 2013). Though Pro and GB do not take part directly in metal-chelation, they stabilize native state of proteins by regulating their water and hence these osmoprotectants (especially Pro) help in maintaining the conformational characteristics and integrity of proteins (Paleg et al., 1981). It may happen due to the capacity of Pro to increase surface tension of water and thus can force water-protein interfaces into contact. This promotes proteins to maintain more native/folded configuration (Arakawa and Timasheff, 1985). Among the amino acids Pro accumulation is generally the highest under metal exposure which may be more than 20-fold in some species like *S. vulgaris* (Sharma and Dietz, 2006). However, their accumulation also varied depending on the metals where the plants were grown. Increased level of Pro was observed in *B. oleracea* in presence of Cd and Hg as reported by Theriappan et al. (2011). The role of Pro in the chelation of metals was reviewed by Sharma and Dietz (2006). One of the important role of Pro is the enhancement of endogenous GSH level from which PCs are synthesized, where the metal binds to the constitutively expressed enzyme PC synthase (PCS), thereby activating it to catalyze the conversion of GSH to PC (Zenk, 1996). Pro-mediated enzyme-protection can be possible via Pro-assisted reduction of free metal ion activity as a result of “metal(Zn/Cd)-Pro-complex” formation (Sharma et al., 1998).

Metal stress tolerance is enhanced by PAs as a result of their multiple roles such as the regulation of endogenous hormones (including IAA; abscisic acid, ABA) and antioxidants or ROS-scavengers (Groppa et al., 2007; Wang et al., 2007; Zhao et al., 2008; Choudhary et al., 2012a). Additionally, a number of PA-gene-expressing transgenic plants have been developed exhibiting enhanced metal-tolerance (Table 1). However, a direct role of PAs in metal-chelation has been least explored. To this end, Put-mediated activation of long-distance Ni-transport within the plant was argued to enhance plant-Ni tolerance (Shevyakova et al., 2011). Spd-induced mitigation of Cr-toxicity was related with reduced Cr-uptake and enhanced titers of PCs (Choudhary et al., 2012b). Put was reported to exhibit a selective effect on ion flux in Cd and Pb exposed plant leaves (Lakra et al., 2006). In many studies, NA was found to play a vital role in Cu-complexation in plants (Takahashi et al., 2003; Kim et al., 2005; Irtelli et al., 2009). Role of NA in the intracellular delivery of metals (and also plant reproductive development) has been reported (Takahashi et al., 2003). In Ni-hyperaccumulator, *T. caerulescens*, NA was found to chelate Ni in the xylem as a response to toxic levels of external Ni, and eventually provide Ni-tolerance (Vacchina et al., 2003). Additionally, NA was reported to perform the chelation and transportation of Fe and few divalent metal ions like Zn, Ni, and Cu in plants (reviewed by Hassan and Aarts, 2011). High levels of Zn, Cd, Cu, Fe, and/or Ni can significantly induce the expression of NA synthase genes (responsible for the synthesis of NA by trimerization of S-adenosylmethionine) (reviewed by Hassan and Aarts, 2011; Mehes-Smith et al., 2013). Over-expression of the *T. caerulescens* NAS3 gene in the Ni-excluder *A. thaliana* was reported to improve its Ni-tolerance and Ni-accumulation in their aerial organs (Pianelli et al., 2005). Similarly, overexpression of the NAS3 gene led to increased accumulation of Fe, Zn, and Cu in *O. sativa* (Kawachi et al., 2009).

Histidine (His) is a major N-donor ligand important for metal chelation in (metal hyperaccumulator) plants. Having carboxyl, amino, and imidazole groups as major structural components, His has been considered as a versatile chelator of metals (such as Ni) in plants (Callahan et al., 2006). Metal-chelation role of free His has been extensively reported in plants exposed to varied metals including Ni (Kerkeb and Krämer, 2003; Richau and Schat, 2009; Richau et al., 2009). Nevertheless, the role of elevated free root cell-His in reduced Ni-vacuolar sequestration and enhanced Ni-xylem loading was evidenced (Richau et al., 2009; Richau and Schat, 2009). To this end, higher free His concentration in roots but less Ni-in root vacuoles were evidenced in *T. caerulescens* (Ni-hyperaccumulator) when compared to *Thlaspi arvense* (non-Ni hyperaccumulator) (Richau et al., 2009). It was advocated that the His-Ni complexes were much less taken up by vacuoles than free Ni ions. Further, an inhibited vacuolar sequestration of “His-Ni complexes” in *T. caerulescens* roots was argued as a result of a higher increase in free His therein (hence an enhanced His-mediated Ni-xylem loading) compared to free Ni in non-Ni-hyperaccumulator *T. arvense* (Richau and Schat, 2009; Richau et al., 2009). In Ni-exposed *Alyssum lesbiacum* and *B. juncea*, Ni uptake was not highly correlated with His uptake but the release of Ni into the xylem was associated with a concomitant release of His from an increased root free His pool (Kerkeb and Krämer, 2003). However, in *Alyssum montanum* (and also in *B. juncea*), these authors evidenced a role of exogenously applied His in conferring enhanced Ni-tolerance possibly as a result of enhanced Ni-flux into the xylem. Enhanced tolerance to excess Ni in transgenic *A. thaliana* overexpressing *StHisG* (*Salmonella typhimurium* ATP phosphoribosyl transferase enzyme) was argued due to the accumulation of about 10-fold higher His level (vs. wild type) (Wycisk et al., 2004). A comprehensive study on the composition of amino acids under Cu stress was done by Irtelli et al. (2009). Among the amino acids His/Pro was found to be the most important Cu chelator in xylem sap of *B. carinata* under Cu toxicity. However, the accumulation of amino acids was dependent on the dose of Cu applied to plants. When *B. carinata* was treated with 5 μM CuSO_4_, the accumulation of His, threonine, Gln, glycine, Pro, and methionine was 140, 22, 7, 23, 6, and 13 μM, respectively which could make about 67, 42, 45, 52, 60, and 33% complexation of 0.94 μmol Cu under a pH of 5.8 (Irtelli et al., 2009). For every case, the accumulation of amino acids was pH dependent. More importantly, in the absence of His, Pro was found to play a very important role in Cu binding and the role of other amino acids (threonine, Gln, glycine, and methionine were negligible in presence of His and Pro (Irtelli et al., 2009). Although other amino acids such as NA was not efficiently involved in the response to excess of Cu but it participates in Cu-transport to the shoots in conditions of deficiency (Irtelli et al., 2009). An amino acid derivative namely 2-amino-3-(8-hydroxyquinolin-3-yl)propanoic acid (HQ-Ala) was reported to form highly stable complexes with most transition metal ions via its metal ion chelating group 8-hydroxyquinoline Lee et al. (2009). Cys can also perform a direct chelation of metals (such as Cd) by synthesizing methionine and GSH/PCs that in turn can sequester metals and provide a higher antioxidant defense in plants (Dominguez-Solis et al., 2004).

## Conclusions and future prospects

Plant tolerance to metal-load-accrued impacts largely decides the efficiency and success of a metal-remediation system (Vangronsveld et al., 2009; Maestri et al., 2010; Anjum et al., 2014a,b,c). Hence, a sound knowledge is essential on the compounds that help plants to keep a tight control over concentrations of free metal(loid)s in cytoplasm. Considering recent reports, this paper attempted to present an orchestrated overview of both thiol- and non-thiol compounds that play significant roles in metal-chelation and maintain low concentrations of free metal(loid)s in cytoplasm. PCs and MTs are among the best characterized –SH compounds that strongly interact with metals, chelate them, reduce their concentrations in cytosol, and finally limit their potential toxicity (Cobbett and Goldsbrough, 2002; Solanki and Dhankhar, 2011; Hossain and Komatsu, 2013). To the other, OAs such as citrate, malate, oxalate, malonate, aconitate, and tartrate, and amino acids and their derivatives including GB, Pro, His, and NA are among non-GSH associated compounds and have also been extensively evidenced to contribute to metal-chelation in plants (Hall, 2002; Sharma and Dietz, 2006; Jakkeral and Kajjidoni, 2011). Both thiol- and non-thiol compounds are functionally related, and may coordinate with other defense system components in order to chelate and/or detoxify and tolerate potential metal impact in plants (Figure 2). The reports appraised herein evidenced extensive studies on thiol-compounds in particular context with metal-tolerance in plants; in contrast, reports on non-thiol compounds is rare. Nevertheless, the literature reviewed herein points toward the need of more molecular-genetic studies in order to get more insights into synthesis pathways, metal-specificity, and biological and non-biological factors responsible for the induction/expression of, and potential coordination among mechanisms underlying thiol- and non-thiol compounds-assisted metal-chelation. Together, the suggested studies will help to develop at large scale the transgenic plants with exceptional capacity to extract, chelate different metals and avert their potential toxic consequences.

### Conflict of interest statement

The authors declare that the research was conducted in the absence of any commercial or financial relationships that could be construed as a potential conflict of interest.
